# Pilot Multi-Omic Analysis of Human Bile from Benign and Malignant Biliary Strictures: A Machine-Learning Approach

**DOI:** 10.3390/cancers12061644

**Published:** 2020-06-21

**Authors:** Jesús M. Urman, José M. Herranz, Iker Uriarte, María Rullán, Daniel Oyón, Belén González, Ignacio Fernandez-Urién, Juan Carrascosa, Federico Bolado, Lucía Zabalza, María Arechederra, Gloria Alvarez-Sola, Leticia Colyn, María U. Latasa, Leonor Puchades-Carrasco, Antonio Pineda-Lucena, María J. Iraburu, Marta Iruarrizaga-Lejarreta, Cristina Alonso, Bruno Sangro, Ana Purroy, Isabel Gil, Lorena Carmona, Francisco Javier Cubero, María L. Martínez-Chantar, Jesús M. Banales, Marta R. Romero, Rocio I.R. Macias, Maria J. Monte, Jose J. G. Marín, Juan J. Vila, Fernando J. Corrales, Carmen Berasain, Maite G. Fernández-Barrena, Matías A. Avila

**Affiliations:** 1 Department of Gastroenterology and Hepatology, Navarra University Hospital Complex, 31008 Pamplona, Spain; jm.urman.fernandez@navarra.es (J.M.U.); maria_rullan@hotmail.com (M.R.); danioyonlara7@hotmail.com (D.O.); bm.gonzalezdelahiguera.carnicer@navarra.es (B.G.); ifurien@yahoo.es (I.F.-U.); juan.carrascosa.gil@navarra.es (J.C.); federico.bolado.concejo@navarra.es (F.B.); luciazabalzasanmartin@gmail.com (L.Z.); juanjvila@gmail.com (J.J.V.); 2 IdiSNA, Navarra Institute for Health Research, 31008 Pamplona, Spain; macalderon@unav.es (M.A.); bsangro@unav.es (B.S.); ai.purroy.lopez@navarra.es (A.P.); isabel.gil.aldea@navarra.es (I.G.); cberasain@unav.es (C.B.); magarfer@unav.es (M.G.F.-B.); 3 National Institute for the Study of Liver and Gastrointestinal Diseases, CIBERehd, Carlos III Health Institute, 28029 Madrid, Spain; jmherranzalzueta@gmail.com (J.M.H.); iuriarte@unav.es (I.U.); galvarez.1@alumni.unav.es (G.A.-S.); mlmartinez@cicbiogune.es (M.L.M.-C.); jesus.banales@biodonostia.org (J.M.B.); marta.rodriguez@usal.es (M.R.R.); rociorm@usal.es (R.I.R.M.); mjmonte@usal.es (M.J.M.); jjgmarin@usal.es (J.J.G.M.); fcorrales@cnb.csic.es (F.J.C.); 4 Program of Hepatology, Center for Applied Medical Research (CIMA), University of Navarra, 31008 Pamplona, Spain; lcolyn@alumni.unav.es (L.C.); mulatasa@unav.es (M.U.L.); 5 Drug Discovery Unit, Instituto de Investigación Sanitaria La Fe, Hospital Universitario y Politécnico La Fe, 46026 Valencia, Spain; leonor_puchades@iislafe.es; 6 Program of Molecular Therapeutics, Center for Applied Medical Research (CIMA), University of Navarra, 31008 Pamplona, Spain; apinedal@unav.es; 7 Department of Biochemistry and Genetics, School of Sciences; University of Navarra, 31008 Pamplona, Spain; miraburu@unav.es; 8 OWL Metabolomics, Bizkaia Technology Park, 48160 Derio, Spain; miruarrizaga@owlmetabolomics.com (M.I.-L.); calonso@owlmetabolomics.com (C.A.); 9 Hepatology Unit, Department of Internal Medicine, University of Navarra Clinic, 31008 Pamplona, Spain; 10 Navarrabiomed Biobank Unit, IdiSNA, Navarra Institute for Health Research, 31008 Pamplona, Spain; 11 Proteomics Unit, Centro Nacional de Biotecnología (CNB) Consejo Superior de Investigaciones Científicas (CSIC), 28049 Madrid, Spain; lcarmona@cnb.csic.es; 12 Department of Immunology, Ophtalmology & Ear, Nose and Throat (ENT), Complutense University School of Medicine and 12 de Octubre Health Research Institute (Imas12), 28040 Madrid, Spain; fcubero@ucm.es; 13 Liver Disease Laboratory, Center for Cooperative Research in Biosciences (CIC bioGUNE), Basque Research and Technology Alliance (BRTA), Bizkaia Technology Park, 48160 Derio, Spain; 14 Department of Liver and Gastrointestinal Diseases, Biodonostia Health Research Institute, Donostia University Hospital, 20014 San Sebastian, Spain; 15 IKERBASQUE, Basque Foundation for Science, 48013 Bilbao, Spain; 16 Experimental Hepatology and Drug Targeting (HEVEFARM) Group, University of Salamanca, Biomedical Research Institute of Salamanca (IBSAL), 37007 Salamanca, Spain

**Keywords:** human bile, cholangiocarcinoma, pancreatic adenocarcinoma, lipidomics, proteomics, machine-learning

## Abstract

Cholangiocarcinoma (CCA) and pancreatic adenocarcinoma (PDAC) may lead to the development of extrahepatic obstructive cholestasis. However, biliary stenoses can also be caused by benign conditions, and the identification of their etiology still remains a clinical challenge. We performed metabolomic and proteomic analyses of bile from patients with benign (*n* = 36) and malignant conditions, CCA (*n* = 36) or PDAC (*n* = 57), undergoing endoscopic retrograde cholangiopancreatography with the aim of characterizing bile composition in biliopancreatic disease and identifying biomarkers for the differential diagnosis of biliary strictures. Comprehensive analyses of lipids, bile acids and small molecules were carried out using mass spectrometry (MS) and nuclear magnetic resonance spectroscopy (^1^H-NMR) in all patients. MS analysis of bile proteome was performed in five patients per group. We implemented artificial intelligence tools for the selection of biomarkers and algorithms with predictive capacity. Our machine-learning pipeline included the generation of synthetic data with properties of real data, the selection of potential biomarkers (metabolites or proteins) and their analysis with neural networks (NN). Selected biomarkers were then validated with real data. We identified panels of lipids (*n* = 10) and proteins (*n* = 5) that when analyzed with NN algorithms discriminated between patients with and without cancer with an unprecedented accuracy.

## 1. Introduction

Human bile is a complex fluid that is produced and secreted by the liver, transported through the bile canaliculi and bile ducts and stored in the gallbladder [1]. In the gallbladder, bile is concentrated approximately by a factor of up to fifteen, and upon feeding it is driven to flow through the common bile duct to be ultimately released into the duodenum [2]. Major roles of bile include the emulsification of dietary lipids and liposoluble vitamins for their digestion and absorption, and the excretion of endobiotics (e.g., bilirubin and cholesterol) as well as xenobiotics (e.g., toxins and drugs). Bile composition reflects its physiological roles, and besides inorganic electrolytes its major components comprise bile acids, phospholipids, cholesterol, bilirubin and a small proportion of proteins [2,3]. The chemical nature and concentrations of the different biliary constituents are influenced by the activity of the cell types that participate in its synthesis, storage and secretion, including hepatocytes, cholangiocytes and gallbladder epithelial cells. In healthy conditions, the concentrations of biliary components are tightly controlled. Therefore, alterations in bile composition may reveal the presence of different hepatobiliary and pancreatic disorders as well as the impairment of enterohepatic circulation [3,4]. Moreover, abnormal bile composition can also contribute to disease progression along the biliary and digestive tracts [3,5,6,7].

The composition of human bile has been studied over decades. Recently, the application of “omic” technologies, mainly based on nuclear magnetic resonance (NMR) spectroscopy and mass spectrometry (MS), has provided a more detailed molecular picture of this fluid [3,4]. A deeper characterization of bile composition may allow not only a better understanding of hepatobiliary physiology, but also the identification of biomarkers to discriminate benign and malignant disease conditions [4,8,9]. Bile is rich in lipids, with bile acids (BAs) accounting for about 72% of the total lipid pool, whereas phospholipids and cholesterol contribute approximately 24% and 4%, respectively [2,10]. BAs, key molecules for dietary fat handling, are mostly conjugated with the aminoacids glycine and taurine. Alterations in BA pool size and composition have been reported in hepatopancreatobiliary diseases [10,11,12,13]. Among biliary phospholipids, the most abundant species (>95%) are phosphatidylcholines (PCs), a broad family of diacylphospholipids with different fatty acid side chains [14,15], while sphyngomyelins (SMs) comprise about 1–3% of total phospholipids [16,17]. PCs, as well as SMs, are important for the emulsification of hydrophobic and potentially cytotoxic BAs, and for the stabilization of mixed micelles involved in excretory functions and fat digestion [1,18]. Changes in total bile PC concentrations also occur in hepatobiliary diseases [11,13,15,19].

Proteins are natural constituents of the biliary fluid, representing about 5% of bile’s dry weight [2]. Proteins may reach the bile from the bloodstream through different cellular pathways, and can also be produced by biliary epithelial cells and hepatocytes [3]. These proteins are thought to play different physiological functions, including immunological defense, biliary protection, lipid transport and enzymatic activities [3]. Changes in the bile proteome also occur in pathological situations, and in some cases such as the formation of gallstones these alterations may contribute to disease progression [20]. The bile proteome may be as well an interesting source of potential biomarkers, since proteins can be released into the bile from diseased cells within the biliary tract or from surrounding organs such as the pancreas [21,22,23,24].

Regarding pancreatobiliary diseases, the accurate etiological diagnosis of biliary stenoses remains a clinical challenge. Strictures of the common bile duct may have a diverse origin [25], and the discrimination between benign and malignant stenoses in early stages has not been satisfactorily achieved yet [26]. Benign conditions include primary sclerosing cholangitis, chronic pancreatitis, choledocolithiasis, bile duct injury and infections, among others. Malignant stenoses are mostly attributable to neoplasias arising from the biliary tree, such as cholangiocarcinoma (CCA) or gallbladder carcinoma, or from the pancreas as in the case of pancreatic ductal adenocarcinoma (PDAC) [26,27,28,29]. CCAs and PDACs are very aggressive neoplasms, and therefore their early diagnosis is essential for the application of potentially curative surgical procedures and/or pharmacological therapies [30,31]. Several diagnostic tools are available to discriminate benign from malignant biliary strictures [29]. These include a range of non-invasive imaging techniques plus endoscopic retrograde cholangiopancreatography (ERCP). ERCP is a commonly applied procedure that allows relief of biliary obstruction in patients with stenosis, while providing high-resolution fluoroscopic images and tissue sampling by biliary brushings and endoluminal biopsies [29]. However, several studies indicate that the sensitivity for malignancy of ERCP, even when combined with brush cytology and fluorescent in situ hybridization, plus the analysis of circulating tumor biomarkes such as carbohydrate antigen 19-9 (CA 19-9), is still far from optimal [29,32,33,34]. Therefore, the identification of new markers that can help in the discrimination between benign and malignant biliary stenoses is very much needed. Interestingly, the ERCP procedure also allows for the collection of biliary fluid in a minimally invasive manner. Taking advantage of this possibility, over the past years a number of studies have performed metabolomic and proteomic analyses of bile obtained from patients with biliary obstruction. Significant alterations in biliary lipid composition, including concentrations of PCs and conjugated BAs [11,12,13,15,19,35,36] or the presence of certain PC oxidized species, could discriminate malignant from benign biliary strictures [37]. Proteomic studies have significantly contributed to the definition of the normal bile composition, adding hundreds of new proteins to the list [22,38,39,40,41,42,43,44]. These proteomic studies have implemented multiple fractionation steps and purification methods of varying complexity prior to MS analyses, and most of them include the evaluation of bile from patients with malignant stenoses due to CCA or PDAC. A number of potential biomarkers that could discriminate malignant disease were identified in these works. If validated in subsequent analyses, the evaluation of a well-selected panel of these biomarkers may increase the diagnostic accuracy of biliary stenosis. On the other hand, bile proteomics can also contribute to a better understanding of the mechanisms of the tumorigenic process [45]. Taken together, these findings reveal the complexity of the bile proteome and attest to the interest of its characterization from both physiological, pathological and diagnostic points of view.

In the present study we have performed parallel metabolomic and proteomic analyses of human bile from patients with benign and malignant (CCA and PDAC) biliary stenoses. For the metabolomic studies, mostly focused on the lipidome of bile, we have implemented MS analysis coupled with ultra-high-performance liquid chromatography (UHPLC-MS). Our platform has a high sensitivity and an unprecedented large coverage of different classes of metabolites with a wide dynamic range [46,47], allowing us to produce a most complete lipidomic profile of human bile. This approach was complemented with a detailed high-performance liquid chromatography (HPLC)-MS/MS profile of biliary BAs and a ^1^H-NMR-based analysis of more hydrophilic metabolites. Our proteomic approach implied a streamlined preparation of the bile samples which leverages the targeted analysis of potential bile protein biomarkers. Data analysis and interpretation in omics-based clinical studies can be challenging. In addition to the intrinsic biological complexity, lack of big cohorts due to limitations in sample gathering or high analytical costs, and intragroup variability of measurements further complicate these studies. In this context, recent works have shown that implementation of artificial intelligence approaches can help to unravel disease-specific markers and pathological mechanisms even in data-limited regimes [48,49,50,51,52]. Therefore, using a novel approach, we have combined metabolomic and proteomic measurements with machine intelligence modeling and synthetic data generation [51,53,54] to identify molecular patterns that can discriminate malignant from benign biliary strictures.

## 2. Results

### 2.1. UHPLC-MS Lipidomic Analysis of Bile

**Figure 1:**
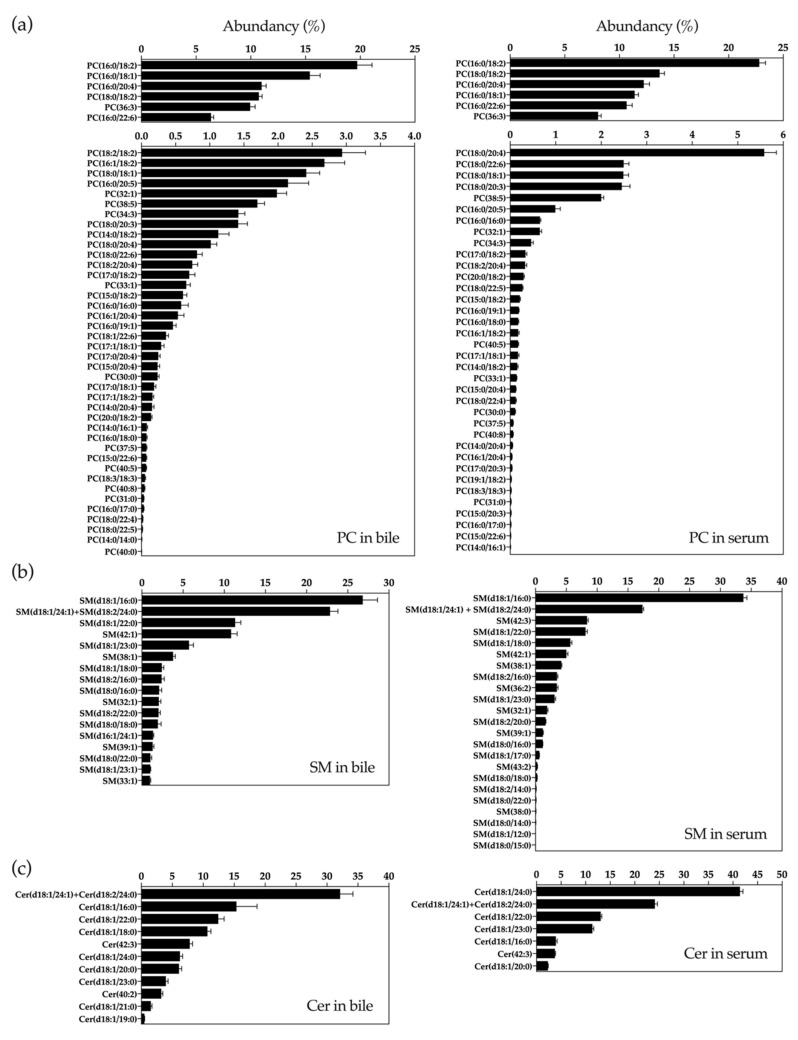
Relative proportions of the different species of phosphatidylcholines (PCs) (**a**) sphingomyelins (SMs) (**b**) and ceramides (Cer) (**c**) found in human bile and serum analyzed by UHPLC-MS (MS analysis coupled with ultra-high-performance liquid chromatography).

**Figure 2:**
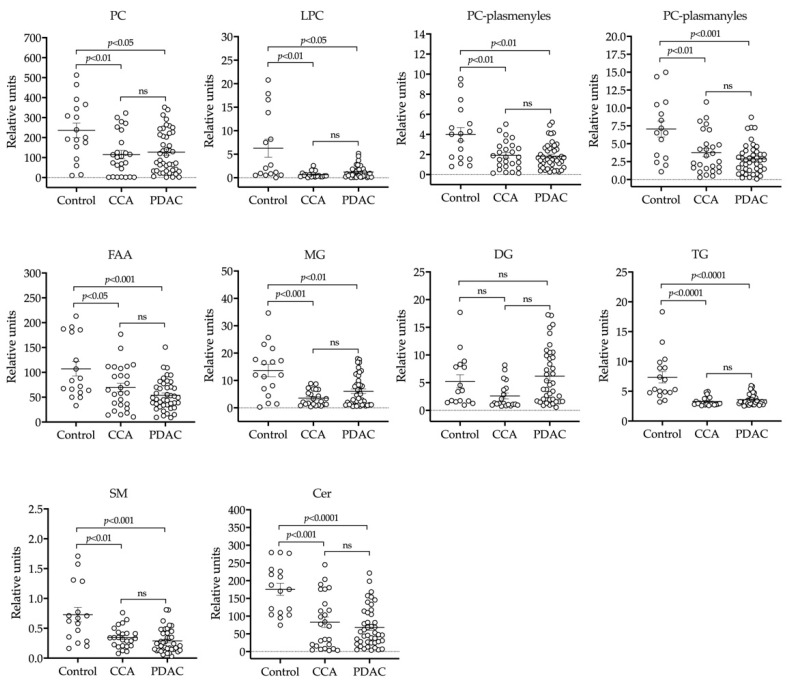
UHPLC-MS-based lipidomic analysis of bile samples from patients with benign stenoses (controls) and patients with CCA (cholangiocarcinoma) or PDAC (pancreatic adenocarcinoma). Lipid species shown include phosphatidylcholines (PC), lysophosphatidylcholines (LPC), PC-plasmenyles, PC-plasmanyles, fatty acid amines (FAA), monoglycerides (MG), diglycerides (DG), triglycerides (TG), sphingomyelins (SMs) and ceramides (Cer).

Bile samples obtained from patients described in Table 1 were processed to extract metabolites with similar lipophilic properties and analyzed in a UHPLC-MS-based platform. We were able to detect 162 metabolic features in these samples belonging to a wide range of lipid species, including fatty acid amines (FAA), monoacylglycerols (MG), diacylglycerols (DG), triacylglycerols (TG), cholesterol (Cho), cholesteryl esters (ChoE), phosphatidyletanolamines (PE), phosphatidylinositols (PI), phosphatidylcholines (PC), phosphatidylcholine plasmanyles and plasmenyles (MEMAPC), lysophosphatidylcholines (LPC), sphingomyelins (SM) and ceramides (Cer). To our knowledge this is the most comprehensive and detailed analysis of the human bile lipidome reported so far. Previous work has found substantial differences in the molecular composition of hepatic and biliary PCs, suggesting the existence of a PC pool destined to biliary secretion [16]. As a large proportion of serum circulating lipids are of hepatic origin, first we decided to compare the bile lipidomic profile of control patients (benign biliary stenoses) with that from our recent analysis of human serum lipidome carried out with the same analytical platform [47]. Because of their high abundance in bile and/or their potential functional significance, we compared the relative contents of the different molecular subspecies of PCs, SMs and Cer detected. As shown in Figure 1a, the six most abundant PC species in bile, which together amounted to over 70% of all PC species, were also the six most abundant species in serum. However, there was more diversity in the next ten most abundant PC subspecies, and their relative proportions were more evenly distributed in bile than in serum. Little is known about the molecular species of SMs and Cer present in human bile. Similar to what was observed for PCs, we found that almost 50% of biliary SMs was accounted for by three highly enriched species, SM(d18:1/16:0), SM(d18:1/24:1) and SM(d18:2/24:0), both in serum and in bile, and albeit in low proportions more SM species were detected in serum (Figure 1b). We detected twelve different molecular species of Cer in bile, with predominance of specific variants such as Cer(d18:1/24:1), Cer(d18:2/24:0), Cer(d18:1/16:0) and Cer(18:1/22:0), metabolically related to the most abundant biliary SMs. We found differences in the relative abundance of some Cer species between bile and serum. For instance, Cer(d18:1/24:0) and Cer(d18:1/23:0) were approximately 7- and 4-fold more abundant in serum, respectively, while Cer(18:1/16:0), the second most-abundant ceramide in bile, was 4-fold less abundant in serum (Figure 1c). Next, we compared the levels of lipid metabolites in bile samples from patients with benign strictures with those in patients with CCA and PDAC-related stenoses (Figure 2). In agreement with previous findings [11,13,15,19,35], we observed an overall reduction in PCs concentrations in bile from CCA and PDAC patients compared to controls. LPCs showed a trend towards reduced levels in samples from CCA, which reached statistical significance in those from PDAC patients. However, the levels of PC plasmanyles and plasmenyles, as well as those of FAAs, were consistently reduced in bile samples from CCA and PDAC patients. Total MGs and TGs were present at lower concentrations in bile form CCA and PDAC patients, while there were no statistical differences in the levels of DGs, which tended to be higher in PDAC patients. As observed for FAAs, total concentrations of SMs and Cer were also reduced in bile from patients with malignant stenoses. We did not observe significant changes in the concentrations of Cho, ChoEs or PEs between control and cancer patients [55]. A heatmap representing all the individual lipid species identified in this analysis, showing their relative levels (fold-change) in bile samples from control vs. CCA and PDAC patients is shown in Table S1.

### 2.2. HPLC-MS/MS Analysis of BAs in Bile

**Figure 3:**
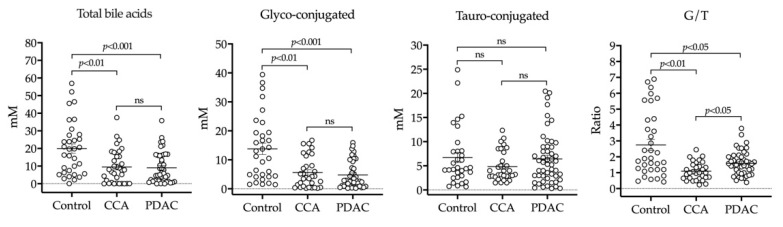
HPLC-MS/MS-based analysis of BAs (bile acids) in bile samples from patients with benign stenoses (controls), CCA or PDAC. Levels of total BAs, glyco-conjugated and tauro-conjugated BAs, along with the ratio between glyco-conjugated and tauro-conjugated species (G/T) are shown.

We also performed a quantitative analysis of BAs in bile samples from our cohort of patients. In agreement with a previous report [15], we found a significant decrease in the total concentrations of BAs in samples from patients with malignant stenoses (Figure 3). Levels of glycine-conjugated BAs, the most abundant species, were reduced in CCA and PDAC samples, while taurine-conjugated BA levels did not change significantly (Figure 3). The ratio of glycine- vs. taurine-conjugated BAs in normal bile is around 3 [56]. Accordingly, in our control bile we found a 2.7 ratio, while in bile samples from CCA and PDAC patients this ratio markedly fell (Figure 3). Previous studies have reported that the concentrations of biliary constituents such as BAs are reduced in bile from patients with biliary obstruction, in an inverse correlation with cholestasis [11,36]. Therefore, we evaluated whether there was a correlation between the total levels of BAs and serum bilirubin or GGT levels in our cohort of patients. Interestingly, a significant negative correlation was found in patients with benign cholangiopathies which was not observed in those with malignant diseases (Figure S1).

### 2.3. H-NMR Analysis of Bile

**Figure 4:**
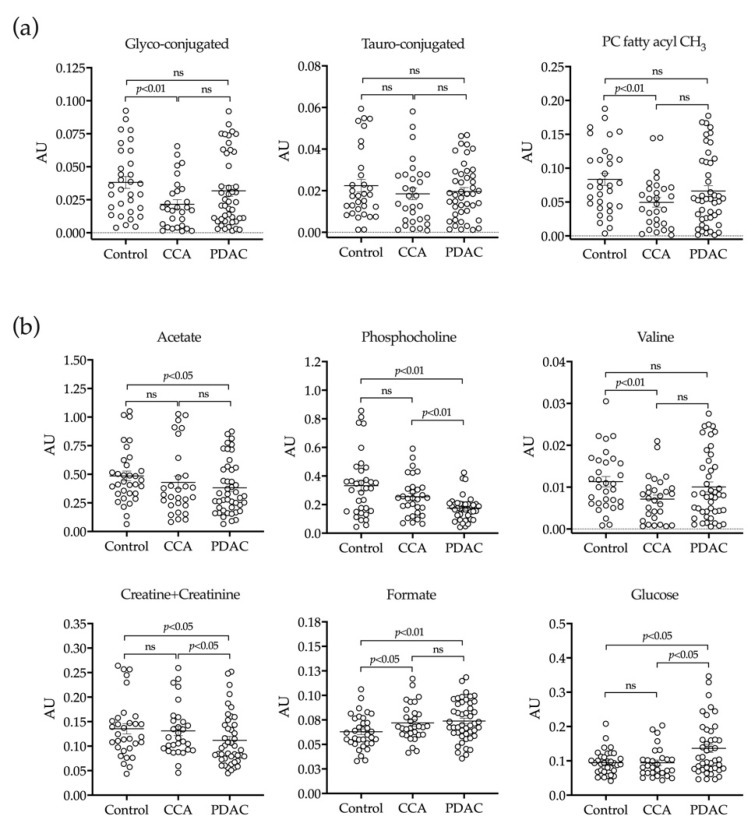
Conjugated BAs and PC (**a**) and water-soluble metabolites (**b**) identified in the ^1^H-NMR-based analysis of bile samples from patients with benign stenoses (controls), CCA or PDAC.

Previous studies evidenced the complex ^1^H-NMR spectrum of human bile, which is due in part to the aggregation of its lipophilic constituents and the overlap of spectral peaks [8]. This complicates the detailed and quantitative evaluation of the bile metabolome unless samples are processed and fractionated prior to their analysis [8]. Our MS-based approaches described above provided a broad and accurate coverage of biliary lipids and BAs. Therefore, bile samples from our cohort were processed to extract more aqueous-soluble metabolites prior to ^1^H-NMR analysis as described in Methods. Spectra were baseline corrected, referenced to the methyl group signal of TSP at 0.00 ppm, aligned and binned into 0.01 ppm wide rectangular buckets over the spectral region δ 8.757–0.261. The residual water (δ 4.78–4.59 ppm) and contrast reagent (Omnipaque) residual (δ 1.98–1.92, 2.43–2.39, 3.71–3.39, 4.18–3.76) signal regions were excluded from further analyses to avoid interference. Nevertheless, the analysis of Omnipaque concentrations in bile samples helped us to rule out a potential confounding effect due to sample dilution. This could alter the concentrations of other metabolites or proteins in bile. In this regard, we did not find any correlation between the concentrations of Omnipaque and BAs, suggesting the absence of a systematic dilution effect of bile samples by contrast reagent [55]. Spectra were then normalized to the total area of the corresponding spectra and by probabilistic quotient normalization (PQN). In our ^1^H-NMR analyses we were able to detect the most hydrophilic conjugated BAs species. We confirmed the reduced levels of glycine-conjugated BAs in bile from CCA patients, and a similar trend in PDAC patients, while taurine-conjugated BAs levels consistently remained unchanged (Figure 4a). In agreement with our MS analysis, the signal corresponding to the PC fatty acyl chain (PC fatty acyl CH_3_) was reduced in bile from CCA patients, and showed a downward trend in bile from PDAC patients (Figure 4a). Interestingly, using this ^1^H-NMR analysis we could detect other water-soluble metabolites whose changes might be related to the pathologic process. These included reduced levels of acetate, phosphocholine, valine and creatine plus creatinine in either CCA or PDAC, but mostly in the latter (Figure 4b). Conversely, formate levels were increased in bile from CCA and PDAC patients, and glucose concentrations were significantly elevated in patients with pancreatic neoplasia (Figure 4b). In view of the high glucose concentrations in bile from PDAC patients, we examined the levels of glycated hemoglobin (HbA1c) in serum, an index of mean glycemia used for the monitoring of long-term glycemic status [57]. Levels of HbA1c found were: 5.93 ± 1.2%, 5.75 ± 1.3% and 6.56 ± 1.4% in controls, CCA and PDAC patients, respectively, and these values were statistically different when data from CCA and PDAC patients were compared (*p* = 0.018).

### 2.4. Application of Machine-Learning Methods to Metabolomic Data to Differentiate between Benign and Malignant Biliary Stenoses

**Figure 5:**
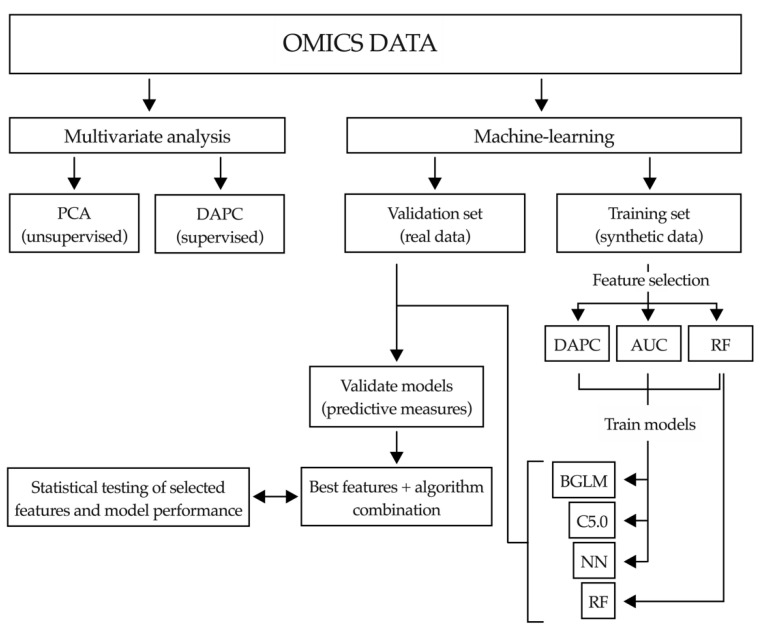
Flowchart of the data analysis. Overview of the methodology for multivariate analysis and machine-learning approach implemented in this study. PCA: principal component analysis; DAPC: discriminant analysis of principal components; AUC: area under the curve; RF: random forest; BGLM: Bayesian variant of general linear model; NN: neural networks.

**Figure 6:**
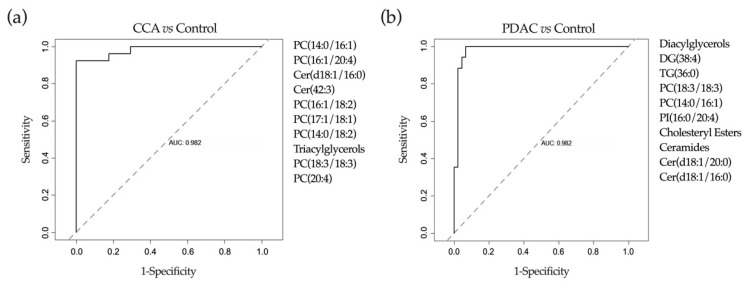
Lipid species present in bile that better predict the presence of malignant stenoses associated with CCA (**a**) or PDAC (**b**) according to machine-learning analyses. Values of AUC are indicated.

Machine learning is a branch of artificial intelligence that when applied in biomedicine can be used to reduce large data sets to small sets of biomarkers with high performance. Machine-learning techniques implement pattern recognition and identify algorithms that can differentiate and predict clinical conditions using complex and non-linearly related data [58]. In view of the complexity of the metabolomic data, in which the number of input variables normally exceeds the number of subjects analyzed [58], we decided to implement machine-learning methods to extract the most useful predictive information. As described in the flowchart presented in Figure 5, first we performed a more conventional multivariate analysis. The unsupervised principal component analysis (PCA) of lipidomic data was not able to discriminate between controls and patients with malignant stenoses [55]. Next, we performed a supervised discriminant analysis of principal components (DAPC), an alternative multivariate method that focus on between-group variability while neglecting within-group variation [59]. This DAPC analysis allows the selection of a set of features, lipid metabolites in this case, which contribute most to the separation between groups (each of them explaining at least 2% of the variability between groups of samples). Their identity, contribution to inter-group variability in the DAPC analysis, Area Under the Curve (AUC) ROC (Receiver Operating Characteristics) curve values, sensitivity and specificity are summarized in Table S2. However, the predictive values of these metabolites, either individually or in combination, was still suboptimal (Table S2). It is becoming evident that to build accurate predictive models applicable in real life large cohorts of patients, then associated data divided into training and validation sets, along with algorithms to identify inner patterns in those data, are necessary. To this end, machine-learning approaches can be very useful. However, for machine-learning tools to work properly, large datasets need to be available. To overcome this situation the generation of synthetic data is gaining interest [48]. The synthetic data has to fulfill two main requisites, on one hand it has to mimic the observations that could be collected from further experiments on each variable, including the “experimental noise”. On the other hand, the data structure has to be maintained. Biological data is full of correlated variables and it is important to maintain that relationship [51]. Once the synthetic data was generated as described in Materials and Methods, we applied three different reduction approaches for feature selection: DAPC, random forest (RF) and AUC analyses. Next, as indicated in Figure 5, the three lists of features selected, including the best three to ten variable combinations, were used to train three different machine-learning algorithms: a Bayesian variant of general linear model (BGLM) [60], C5.0 [61] and neural networks (NN) [62]. In the case of RF, it was only challenged with its own list, with the purpose of comparing it as a gold standard algorithm for this study [63]. Once trained, real data was used to validate the predictive capacity of the algorithms. With this approach we were able to select the best feature combination and the algorithm with higher predictive capacity for that set of features. We found that optimal feature selection and predictive performance was obtained with the combination of DAPC (top ten features) and NN analysis. The robustness of this model was evaluated with five statistical tests as described in Materials and Methods. These tests assessed the accuracy in feature selection (influenced by the number of samples), the impact of the inclusion or exclusion of each selected feature in the analysis, the existence of the inner pattern of the data identified by the algorithm, the analysis of the contribution of each individual variable to the performance of the model, and whether the model was or not overfitted and prone to detect artificial patterns. With this approach we identified a combination of lipid species (features) that when analyzed with the NN algorithm (structure of this neural network is shown in Figure S2a) permitted us to differentiate between patients with benign stenoses and CCA with an AUC of 0.984, 94.1% sensitivity and 92.3% specificity. These species, all described in the bile lipidomic analysis provided in Table S1, encompassed a series of PCs, including those containing arachidonic acid (20:4), certain Cers and total TGs levels (Figure 6a). With a similar approach, we identified a combination of lipid species that when analyzed with the NN algorithm (structure of the neural network is shown in Figure S2b) could differentiate control patients from those with PDAC with an AUC of 0.98, 88% sensitivity and 100% specificity. These lipids, also described in Table S1, included PCs, two specific Cer, DG and TG species, plus the total levels of Cers, cholesteryl esters, the total levels of DGs and a phosphatidylinositol (Figure 6b). As reported above, we also performed a metabolomic analysis using an ^1^H-NMR platform and a detailed evaluation of the BAs profile. Therefore, we tested whether the inclusion of these data sets in our machine-learning pipeline could improve the performance of the model. However, the incorporation of this information in the analysis did not provide any advantage and neither did the inclusion of serum CA 19-9 levels [55].

### 2.5. Proteomic Analysis of Bile

**Figure 7:**
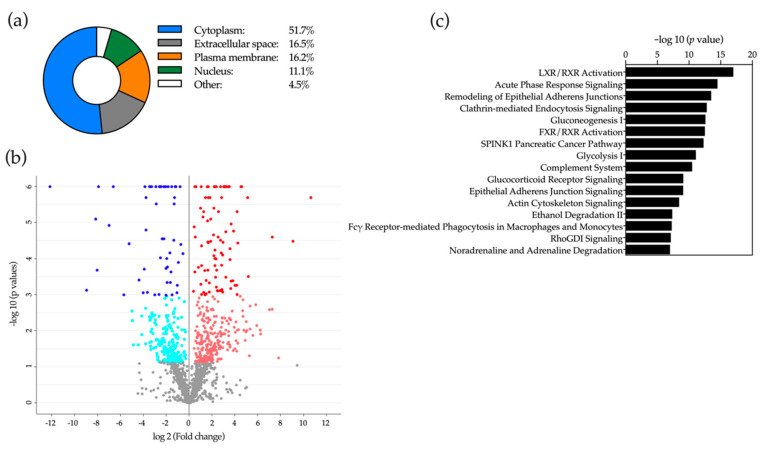
Proteomic analysis of bile from patients with benign stenoses and patients with CCA. (**a**) Pie chart showing the classification of proteins according to their cellular localization. (**b**) Volcano plot (−log10 [*p*-value] and log2 [fold-change]) of the proteins found in bile from patients with CCA compared with patients with benign stenoses. (**c**) Ingenuity pathway analysis (IPA) of the differentially represented proteins between control and CCA bile samples identifying the top enriched categories of canonical pathways.

**Figure 8:**
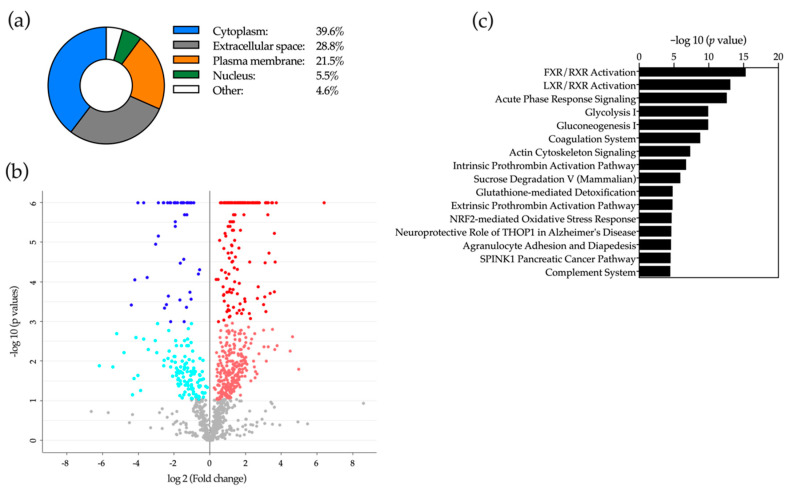
Proteomic analysis of bile from patients with benign stenoses and patients with PDAC. (**a**) Pie chart showing the classification of proteins according to their cellular localization. (**b**) Volcano plot (−log10 [*p*-value] and log2 [fold-change]) of the proteins found in bile from patients with PDAC compared with patients with benign stenoses. (**c**) Ingenuity pathway analysis (IPA) of the differentially represented proteins between control and PDAC bile samples identifying the top enriched categories of canonical pathways.

Next, we performed two independent LC-MS based proteomic analyses of selected bile samples. Two sets of samples were used, one obtained from control patients with benign cholangiopathy (*n* = 5) and CCA patients (*n* = 5), and another set from a second group of control patients with benign cholangiopathy (*n* = 5) and from patients with PDAC (*n* = 5). In the first experiment we identified a total of 2042 proteins, most of them of intracellular origin: nucleus, cytoplasm and plasma membrane (Figure 7a). Of these proteins, 387 were found upregulated and 243 were downregulated in samples from CCA patients compared to controls (Figure 7b and Table S3). Ingenuity pathway analysis (IPA) of the differentially represented proteins in bile from patients with benign conditions and from CCA patients allowed their preferential classification in certain biological processes (Figure 7c). In agreement with previous proteomic studies [3,9,24,43,64], the canonical pathways enriched in our IPA analysis identified categories such as inflammation (acute phase response and complement), metabolic regulation by nuclear receptors of BAs and sterols, glucose metabolism, tissue architecture (cell-cell interactions), oxidative stress and cell signaling. The identity of many of these proteins, both upregulated and downregulated (Table S3), is consistent with previously published observations [9,21,43,64,65,66]. When we analyzed bile samples from patients with benign cholangiopathy and PDAC, we identified a total of 1115 proteins. The cellular distribution was similar to that observed in the previous analysis, although the proportion of proteins of cytoplasmic origin was reduced while that of proteins belonging to the extracellular space was increased compared to bile samples from CCA patients (Figure 8a). Among these proteins, 410 were upregulated in bile samples from PDAC patients, while 123 were downregulated (Figure 8b, Table S4). IPA analysis of the differentially expressed proteins identified a series of enriched canonical pathways that overlapped to a great extent with those found in the analysis of bile samples from benign conditions and CCA (Figure 8c). A significant number of the proteins identified in our study (Table S4) were consistent with previous reports that analyzed the bile proteome from patients with PDAC-related stenoses [3,21,42,67,68].

### 2.6. Application of Machine-Learning Methods to Bile Proteomic Data to Differentiate between Benign and Malignant Stenoses

For the analysis of the proteomic data and to identify proteins that could discriminate malignant stenoses we followed the same approach depicted in Figure 5. As found in the lipidomic study, unsupervised PCA analysis did not discriminate between controls and patients with CCA-related stenoses (Figure S3a). Next, we performed a supervised DAPC analysis that allowed the selection of a set of features, proteins, which contributed most to the separation between groups (each of them explaining at least 2% of the variability between groups of samples). Their identity, up or downregulation, magnitude of change between control and CCA samples and contribution to inter-group variability according to the DAPC analysis are summarized in Figure S3b. An equivalent analysis was performed with the proteomic data obtained from a different set of bile samples from control and patients with PDAC-related malignant stenoses. Unsupervised PCA analysis was not able to discriminate between groups (Figure S3c). As for the CCA samples, the application of DAPC analysis selected a set of proteins that contributed most to the separation between groups. Their identity, variations in control vs. PDAC bile samples and contribution to intergroup variability are presented in Figure S3d.

The great majority of the proteins selected in the DAPC analysis have been previously detected in human bile [22,38], and many of them are also known to be altered in hepatobiliopancreatic malignancies. For instance, alpha-2-macroglobulin (A2M) and alpha-4-actinin (ACTN4), both selected among the upregulated proteins in our analysis of CCA bile, are known to be increased in bile [43] and tissues [69] from CCA patients, respectively. Phosphoglycerate kinase 1 (PGK1), an essential enzyme in aerobic glycolysis elevated in tumors and serum from cancer patients [70], has not been previously found in bile. However, sucrase-isomaltase (SI), an intestinal mucosa α-glucosidase [71] was previously detected in human bile [21] but not related to cancer. Among the downregulated proteins we detected carboxypeptidase M (CPM), 5′-nucleotidase (NT5E), myeloperoxidase (MPO), lactotransferrin (LTF) and desmoplakin (DSP), all of them previously found in human bile [22,38] with the exception of LTF. Interestingly, in the proteomic analysis of bile from patients with PDAC the DAPC analysis identified a different set of discriminant proteins. Some of them, such as albumin (ALB) and apolipoprotein B-100 (APOB), have also been previously reported as more abundant in bile from PDAC patients [43]. Mucin 5B (MUC5B), a little-characterized secretory type of mucin previously found in human bile and overexpressed in PDAC tissues [22,72], was also selected. Interestingly, two other proteins identified in this analysis were the PC transporter ABCB4 (MCP3) and the angiotensin converting enzyme 2 (ACE2), both known to be upregulated in PDAC tissues [73,74]. Finally, among the proteins selected by the DAPC analysis that were less abundant in bile from these patients were pancreatic alpha-amylase (AMY2A), previously found in bile [38], ectonucleotidase pyrophosphatase/phosphodiesterase 7 (ENPP7), also known as alkaline sphingomyelinase (alk-SMAse), which is less abundant in bile from patients with pancreatobiliary malignancies [75], and protocadherin fat 4 (FAT4), a presumed tumor suppressor gene frequently mutated and silenced in solid tumors [76].

**Figure 9:**
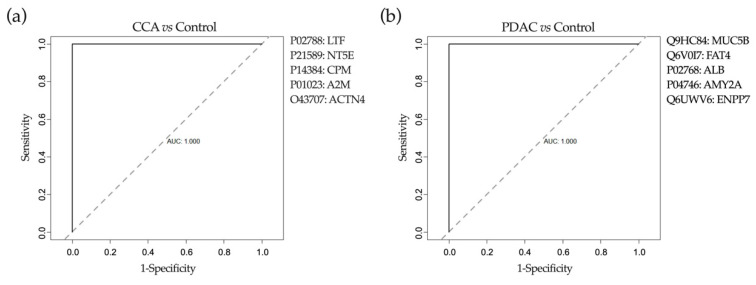
Identity of the proteins present in bile that better predict the presence of CCA (**a**) or PDAC (**b**) malignant stenoses. Values of AUC are indicated.

Altogether, the DAPC analysis identified potential candidate proteins to discriminate between patients with benign and malignant pathologies. Nevertheless, and as stated before, to build robust predictive models larger cohorts of patients together with algorithms that identify inner data patterns and interrelationships are necessary. Therefore, we implemented the same machine-learning approach used for the lipidomic analysis (Figure 5). After synthetic data was generated we applied on it three different reduction approaches for feature selection: DAPC, RF and AUC analysis. Next, and as indicated in Figure 5, the three lists of features selected, including the best three to ten variable combinations, were used to train three different machine-learning algorithms: BGLM, C5.0 and NN. We identified a combination of five proteins (features) that when analyzed with the NN algorithm (structure of the neural network is shown in Figure S4a) and validated with the real data set performed best. It permitted us to differentiate between patients with benign cholangiopathy and CCA with an AUC of 1, 100% sensitivity and 100% specificity (Figure 9a). Similarly, five proteins were identified that when analyzed with the NN algorithm (structure of the neural network is shown in Figure S4b) allowed the discrimination between control and PDAC patients with an AUC of 1, 100% sensitivity and 100% specificity (Figure 9b). As observed before for the lipidomic study, the features identified by the DAPC analysis of the real data also overlapped to some extent with those selected by the DAPC analysis of the synthetic data.

## 3. Discussion

In our lipidomic analysis were able to identify more than 45 molecular species of PC in human bile. In agreement with previous studies, the most abundant PC species had a 16:0 moiety in the *sn*-1 position and an unsaturated acyl chain (18:1, 18:2, 20:4) in the *sn*-2 position, and these species were followed by those with a *sn*-1 18:0 moiety [15,16,77]. The relative composition of PC species found in normal human serum, as we previously described using this same analytical platform [47], was similar. Our findings confirm previous studies indicating the selection of the least hydrophobic types of lecithins from the hepatic pool for biliary secretion [16,77]. Regarding SMs, we identified up to 18 species in bile, with an enrichment in d18:1/16:0 SM, the least hydrophobic molecular species, as previously reported for rat bile [17]. As observed for PCs, the most abundant SM species in bile were also found among the most abundant in serum. Observations in experimental and in vitro models indicate that the presence of d18:1/16:0 SM in bile may contribute to canalicular bile formation [17,78]. Our findings suggest that the relatively high abundance of d18:1/16:0 SM may also contribute to bile formation in humans. There is little information available on the presence and function of Cer in bile. Cer are biosynthetically related to both SMs and PCs, and are widely recognized as potent active lipids controlling many aspects of cell biology, from survival and proliferation to the regulation of metabolism [79,80]. We identified 12 different species of Cer. At variance with the relative conservation of PCs and SMs species between bile and serum, the relative abundance of Cer types was more diverse. Interestingly, the most abundant Cers in bile (almost 50% of total Cer) were Cer (d18:1/24:1), Cer(d18:2/24:0) and Cer(d18:1/16:0), which can be produced by the action of sphingomyelinases, such as alk-SMase present in human bile [81,82], on SM(d18:1/24:1), SM(d18:2/24:0) and SM(d18:1/16:0), which in turn are the most abundant SMs in bile. Our findings on the levels of the most abundant SMs and Cers in bile and serum are generally in agreement with a recent study that analyzed these metabolites in human serum [83]. Very long chain Cer species, such as Cer(d18:1/24:1) and Cer(d18:2/24:0), have been reported to display cytoprotective properties [84]. Their relative enrichment in bile could have a protective role towards the biliary epithelium.

Next, we compared the relative contents of the major types of lipids present in bile samples from patients with benign stenoses and from patients with CCA or PDAC. In agreement with previous reports, we observed a reduction in the total levels of PC in patients with malignant stenoses [13,35,85,86]. This was accompanied by a reduction in total MGs and TGs levels. The reason for reduced PC concentrations in bile from patients with malignant strictures is not well understood. Malnutrition, often present in patients with biliopancreatic tumors, could account for the reduced contents of PC and glycerolipids, and indeed the PNI, an index of nutritional status [87], was slightly lower in CCA and PDAC patients. However, cholesterol levels were not different among groups, and DG contents tended to be higher in PDAC patients. Impaired secretion of PC into bile has been proposed as a potential explanation [13,35]. PC secretion is dependent on the hepatocyte membrane flippase multidrug resistance protein 3 (MDR3, *ABCB4* gene) [88]. Decreased expression of *ABCB4* has been found associated with liver inflammation [89]. The inflammatory environment that accompanies hepatobiliary tumorigenesis [90] could hypothetically result in downregulation of *ABCB4* expression, as occurs for other hepatocellular membrane transporters [91], however this contention needs to be directly addressed. Interestingly, the presence of high SM levels in the canalicular membrane of hepatocytes seems to be essential for optimal MDR3 function and PC efflux [92]. We found that the levels of SMs, along with those of Cer, were also lower in bile from patients with neoplastic disease. In view of the positive influence of SM on PC secretion, reduced SM availability in parenchymal cells might also contribute to impaired PC release into bile. Alternatively, increased hydrolysis of PC by phospholipases has been proposed as a possible mechanism [36], which would be consistent with the enhanced metabolism of choline phospholipids in cancer tissues [93]. However, the reduction in SM contents might not be attributable to its enhanced degradation, as the levels of alk-SMase are markedly downregulated in bile from patients with pancreatobiliary malignancies [82], as we also found. Levels of ether glycerophospholipids, both plasmanyles and plasmenyles, were also lower in bile from patients with CCA and PDAC. Plasmenyles, also known as plasmalogens, were particularly reduced. Plasmalogens are secreted from the liver in lipoproteins. Due to their reactivity with free radicals, and in a process that entails their degradation, these lipid species play an antioxidant role in plasma [94]. The presence of plasmalogens in bile suggests that they could also have an antioxidant role in this fluid. On the other hand, the lower levels of plasmalogens in bile from patients with CCA and PDAC might be due in part to the pro-oxidative and inflammatory conditions associated with neoplasia [3,37].

Our study included the analysis of BA levels in bile. We found a significant reduction in the total concentrations of BAs in patients with malignant stenoses. In healthy adults the majority of BAs in bile are conjugated with glycine and taurine in a proportion close to 3:1 [56]. In agreement with previous reports [15,56], our data were consistent with this concept. Furthermore, we observed a reduction in the total concentrations of BAs in patients with malignant disease, which was mainly due to a decrease in glycine-conjugated species. Contrary to our findings, other works have reported an increase in glycine-conjugated BAs levels in bile from CCA patients [13,86]. The reason for this discrepancy is not known. It could be related to the fact that the patients included in those studies might be at more advanced stages of the disease than those in our cohort. Reduction in bile constituents has been associated with biliary obstruction, an increase in the back pressure on the liver during cholestasis and enhanced regurgitation into serum of bile constituents, such as BAs and bilirubin [11,36]. However, in our CCA and PDAC patients, we did not find any inverse correlation between levels of BAs in bile and bilirubin in serum. The reduction in biliary BAs in these patients could be related to mechanisms more specifically associated with the neoplastic process. For instance, it is known that the expression of the canalicular export pumps MRP2 and the bile salt export pump (BSEP) is markedly reduced by inflammatory cytokines, including tumor necrosis factor α (TNFα) [91], which are abundant in the malignant biliary microenvironment [3,4].

The ^1^H-NMR analyses partially confirmed our previous LC-MS-based findings on the reduced levels of glycine-conjugated BAs and PC in bile from patients with malignant strictures. Furthermore, we identified a series of hydrophilic small molecules such as acetate, phosphocholine, valine and creatine/creatinine, with concentrations reduced mostly in bile from PDAC patients. Some of these differences may indeed be attributed to the presence of an ongoing malignant process. For instance, tumor cells have been shown to capture acetate as a carbon source to sustain growth [95]. In addition, the turnover and usage of choline metabolites like phosphocholine, branched chain amino acids such as valine, and energy-storing molecules like creatine are known to be markedly altered in neoplastic tissues [96,97]. Similarly, the rise in formate levels detected both in CCA and PDAC patients can be linked to the hyperactivity of a myriad of metabolic pathways related to one carbon metabolism which are essential for cell growth, such as polyamine and purine synthesis, in which formate is produced in excess and can be released from cells [98]. Taken together, these changes in bile metabolome may represent the microenvironmental footprint of the profound rewiring of metabolism that drives tumorigenesis [99,100]. Most interestingly, and only in PDAC patients, we also detected a significant increase in the levels of glucose. This finding was somehow puzzling, as tumor cells avidly uptake glucose from the extracellular milieu [100]. However, previous reports described an association between disturbances in glucose metabolism in the absence of a history of diabetes and the presence of PDAC [101,102]. Consistently, we found that the serum levels of HbA1c, a marker of glycemic status, were selectively elevated in PDAC patients. These findings suggest that elevated glucose levels in bile may be associated with the presence of pancreatic malignancies.

The second aim of this work was to select molecular features (metabolites and proteins) identified in bile that could be applied for the discrimination between patients with benign and malignant strictures. However, on one hand, clinical samples tend to show high complexity and variability in their molecular composition even among same groups of patients, and on the other hand, “omics” studies are still costly to perform, a factor that limits the availability of data. It is likely that these circumstances have hampered the identification of robust biomarkers with diagnostic value for many diseases, including the discrimination between benign and malignant biliary strictures addressed in our study. To circumvent these issues, first we implemented a relatively new multivariate method known as DAPC, until now mostly used in the field of genetics, that can detect hidden and non-trivial biological patterns and define groups or clusters of individuals [59]. This analysis identifies the features (metabolites or proteins in our case) that mainly contribute to the separation (variability) between groups with great accuracy. In spite of this, given the high variability commonly found in clinical samples, the direct quantitation of these features may not be sufficient for their precise adscription to a specific group, i.e., healthy or diseased. However, the complex and nonlinear relationships that exist between features may give rise to additional patterns that may be used to generate models with predictive capacity when applied to new sets of data. These patterns can be detected by implementing machine-learning approaches [48]. However, the majority of machine-learning methods require data sets that are orders of magnitude larger than those gathered in “omics” studies with limited number of patients. This is why we decided to augment our data set with computer-generated and artificially noised data to train different deep learning algorithms [48,51]. Using this approach with bile lipidomic data we selected two sets of features, lipid species, that when analyzed with NN allowed a very good separation between control patients and those with CCA or PDAC-related strictures. Interestingly, the lipids selected by our DAPC and NN algorithm as the most sensitive biomarkers were not among the most abundant species present in bile, or those that experienced the most dramatic changes. A similar observation has been recently made in a machine-learning-driven lipidomic study analyzing serum sphingolipids to define markers of cardiovascular disease. The best performing biomarker panel identified mainly comprised the less abundant SMs and Cers present in serum [52].

Our proteomic analyses also implemented an equivalent synthetic data generation approach, DAPC-based selection of features and machine-learning pipeline. We have identified a reduced panel of proteins that upon NN analysis provided accurate separation between patients with benign and malignant stenoses. As mentioned before, the identity and nature of the alterations (up or downregulation) of some of these proteins could have biological significance regarding the evolution of the malignant processes. For instance, LF, which is downregulated in bile from CCA patients, has been described as a cytoprotective factor for cholangiocytes, and therefore its reduction may contribute to cell injury, death and inflammation [103]. Conversely, ACTN4, which was upregulated in bile from CCA patients, has been reported as a crucial factor for the progression of a variety of solid tumors [104]. Similarly, MUC5B, more abundant in bile from PDAC patients, has been described to contribute to the survival and migration of pancreatic cancer cells [72]. However, FAT4, which is reduced in bile from these patients, is a cadherin-related protein identified as a tumor suppressor in gastric cancer [105]. Altogether, these findings may provide new mechanistic insights into pancreatobiliary carcinogenesis. Nevertheless, similar to our findings in our lipidomic study, it is worth noticing that the proteins selected here as biomarkers were not among those proteins that underwent major changes in their relative abundance between controls and patients with malignant disease. These observations further attest to the potential of machine-learning tools for biological data mining and the selection of clinically informative patterns.

## 4. Materials and Methods

### 4.1. Patient Population and Samples Collection

A cohort of 129 patients prescribed to undergo ERCP with a diagnosis of bile duct stenosis (*n* = 104) or choledocholithiasis (*n* = 25) was prospectively accrued for the study from January 2017 to December 2019 at the Navarra University Hospital Complex. All patients were older than 18 years and provided written informed consent for the examination of their samples and the use of their clinical data. Patients with clinical or analytical data of cholangitis at the time of ERCP were excluded. The study protocol was approved by the Ethics Committee of the Navarra University Hospital Complex (protocol # 2016/91).

The tumoral origin of the biliary stenosis was obtained after a pathological diagnosis (*n* = 76) or, failing that, after a clinical diagnosis (*n* = 17), which was established in the presence of imaging tests of a mass that strictures the bile duct without the presence of acute cholangiopathy, together with a clinical or radiological progression after 12 months of follow-up or death related to neoplastic disease, as described in other related studies [33]. A total of 11 patients with biliary stenosis presented a resolution or stability of the same after more than 12 months of clinical and radiological follow-up. The cause of these biliary stenoses was related to benign cholangiopathy (*n* = 9) or chronic pancreatitis (*n* = 2). The demographic and clinical characteristics of the patients are summarized in Table 1.

Patients were fasted overnight and ERCPs were conducted in a specific room by highly experienced endoscopists. During standard ERCP procedure, after cannulation of the bile duct, and in most cases before contrast injection (Omnipaque, iohexol), a bile sample of 2 to 6 mL from each patient was aspirated through the sphincterotome. In cases of biliary stenosis, the sample was taken from the bile duct proximal to biliary stenosis and in cases of choledocholithiasis, the sample was taken when the tip of sphincterotome was in the lower third of the common bile duct, which was confirmed under fluoroscopy. After collection bile samples were maintained at 4 °C, centrifuged for 10 min (4 °C) at 3500 *g* and stored in aliquots at −80 °C in our biobank facility. All the process was performed in less than 2 h. Serum samples from all patients were also obtained at the time of ERCP and stored at −80 °C.

### 4.2. Lipidomic Analyses

#### 4.2.1. Lipid Extraction and Uhplc-Ms Analysis

Bile samples were mixed with sodium chloride (50 mM) and chloroform/methanol (2:1) in 1.5 mL microtubes at room temperature. The extraction solvent was spiked with metabolites not detected in unspiked human bile samples [SM(d18:1/6:0), PE(17:0/17:0), PC(19:0/19:0), TG(13:0/13:0/13:0), Cer(d18:1/17:0) and ChoE(12:0)]. After brief vortex mixing, the samples were incubated at −20 °C for 1 h. After centrifugation at 16,000 *g* for 15 min, the organic phase was collected and dried under vacuum. Dried extracts were then reconstituted in acetonitrile / isopropanol (1:1), centrifuged (18,000 *g* for 5 min) for analysis.

Extracts were analyzed by ultra-high-performance liquid chromatography (UHPLC)-time of flight (ToF)-mass spectrometry (MS). Chromatographic and spectrometric conditions were as previously described [46,47]. This analysis provided coverage over glycerolipids, cholesterol esters, sphingolipids and glycerophospholipids.

#### 4.2.2. Lipidomics Data Analysis

Data were pre-processed using the TargetLynx application manager for MassLynx 4.1 software (Waters Corp., Milford, CT, USA). Metabolites were identified prior to the analysis. Peak detection, noise reduction and data normalization were performed as previously described [106].

### 4.3. Analysis of BAs

BA concentrations in bile were measured by the 3α-hydroxysteroid dehydrogenase method. Bile acids were extracted and analyzed by high performance liquid chromatography-tandem mass spectrometry (HPLC-MS/MS) using a 6420 Triple Quad LC/MS (Agilent Technologies, Santa Clara, CA, USA) as we previously reported [107,108].

### 4.4. H-NMR Analysis

#### 4.4.1. Sample Preparation

Frozen bile samples were placed on ice and allowed to thaw for 5 min. Then, 600 µL of chloroform/methanol (2:1, v/v) at 4 °C was added. Samples were homogenized with a vortex and incubated on ice for 10 min. Then, samples were centrifuged at 10,000 *g* for 30 min at 4 °C to allow phase separation. The aqueous phase was transferred to a different tube and lyophilized overnight to remove water and methanol. Samples were stored at −80 °C until NMR sample preparation and measurement.

At the time of ^1^H-NMR analysis, samples were placed on ice and allowed to thaw for 5 min. 600 µL of deuterated water containing 0.5 mM trimethylsilylpropionic acid-d4 sodium salt (TSP), as internal standard, were added to the samples. The samples were vortexed and then centrifuged at 10,000 *g* for 5 min and 550 µL of the supernatant was transferred into a 5 mm NMR tube for analysis.

#### 4.4.2. H-NMR Experiments and Metabolite Quantification

NMR measurements were acquired using an NMR Bruker AVANCE-TM 600 MHz Spectrometer with a 5 mm BBI probe, the acquisition temperature was set at 37 °C. A one-dimensional (1D) NOESY pulse sequence [109] was collected for each sample with 256 scans and 65 K data points over a spectral width of 20 ppm. A 4-s relaxation delay was included between free induction decays (FIDs). Finally, all spectra were automatically phased, baseline corrected, and referenced to the methyl group signal of TSP at 0.00 ppm using TopSpin 3.5 (Bruker Biospin, Rheinstetten, Germany).

For metabolite quantification, after acquisition, NMR signals were integrated and quantified using NMRProcFlow v.1.2.28 [110]. NMRProcFlow is an open source software for data processing prior to multivariate statistical analysis, including, among other tools, solvent signal suppression, internal calibration, phase, baseline and misalignment corrections, bucketing and normalization. Briefly, spectra were binned into 0.01 ppm wide rectangular buckets. The residual water and Omnipaque signal regions were excluded from further analyses to avoid interferences. Spectra were then aligned, normalized to the total area of the corresponding spectra and by probabilistic quotient normalization (PQN) [111]. Metabolites of interest were assigned using Bruker NMR Metabolic Profiling Database BBIOREFCODE 2.0.0 database (Bruker Biospin), in combination with other existing public databases [112,113]. All detectable NMR signals were integrated for further analysis.

### 4.5. Proteomic Analyses

#### 4.5.1. Sample Preparation

Protein digestion in the S-Trap**^TM^** filter (Protifi, Huntington, NY, USA) was performed following the manufacturer’s procedure with slight modifications. Briefly, 30 µL of bile was first mixed with 5% SDS and 5 mM TCEP (final concentrations), reduced at 37 °C for 60 min, followed by addition of 1 µL of 200 mM cysteine-blocking reagent MMTS (SCIEX) for 10 min at room temperature. Afterwards, 12% phosphoric acid and then seven volumes of binding buffer (90% methanol; 100 mM TEAB) were added to the sample (final phosphoric acid concentration: 1.2%). After mixing, the protein solution was loaded to an S-Trap**^TM^** filter in two consecutive steps, separated by a 2 min centrifugation at 3000 *g*. Then the filter was washed 3 times with 150 μL of binding buffer. Finally, 1.5 µg of MS-grade trypsin was added to a 100 mM TEAB solution and spun through the S-Trap prior to digestion. Flow-through was then reloaded to the top of the S-Trap**^TM^** column and allowed to digest o/n at 37 °C. To avoid liquid leakage from the S-Trap**^TM^** column, a customized yellow tip with 9 Empore 3M C18 disks (Sigma-Aldrich, St. Louis, MO, USA) was placed at the bottom tip of the S-Trap column during digestion. To elute peptides, two step-wise buffers were applied (1) 40 μL of 25 mM TEAB and 2) 40 μL of 80% acetonitrile and 0.2% formic acid in H_2_O), separated by a 2 min centrifugation at 3000 *g* in each case. Eluted peptides were pooled and vacuum centrifuged to dryness.

#### 4.5.2. LC-MS Analysis

Digested samples were cleaned-up/desalted using SEP-PAK C18 cartridges (Waters, Milford, MA, USA). After desalting, peptide concentration was carried out by Qubit™ Fluorometric Quantitation (Thermo Fisher Scientific, Waltham, MA, USA). A 1 µg aliquot of each digested sample was subjected to 1D-nano LC-ESI-MS/MS analysis using a nano liquid chromatography system (Eksigent Technologies nanoLC Ultra 1D plus, SCIEX, Foster City, CA, USA) coupled to high speed Triple TOF 5600 mass spectrometer (SCIEX, Foster City, CA, USA) with a Nanospray III source. The analytical column used was a silica-based reversed phase Acquity UPLC^®^ M-Class Peptide BEH C18 Column, 75 µm × 150 mm, 1.7 µm particle size and 130 Å pore size (Waters). The trap column was a C18 Acclaim PepMap^TM^ 100 (Thermo Scientific), 100 µm × 2 cm, 5 µm particle diameter, 100 Å pore size, switched on-line with the analytical column. The loading pump delivered a solution of 0.1% formic acid in water at 2 µL/min. The nano-pump provided a flow-rate of 250 nL/min and was operated under gradient elution conditions. Peptides were separated using a 250 min gradient ranging from 2% to 90% mobile phase B (mobile phase A: 2% acetonitrile, 0.1% formic acid; mobile phase B: 100% acetonitrile, 0.1% formic acid). Injection volume was 5 µL.

Data acquisition was performed with a TripleTOF 5600 System (SCIEX, Foster City, CA, USA). Data were acquired using an ion-spray voltage floating (ISVF) 2300 V, curtain gas (CUR) 35, interface heater temperature (IHT) 150, ion source gas 1 (GS1) 25, declustering potential (DP) 100 V. All data were acquired using information-dependent acquisition (IDA) mode with Analyst TF 1.7 software (SCIEX). For IDA parameters, 0.25 s MS survey scan in the mass range of 350–1250 Da were followed by 35 MS/MS scans of 100 ms in the mass range of 100–1800 (total cycle time: 4 s). Switching criteria were set to ions greater than mass to charge ratio (m/z) 350 and smaller than m/z 1250 with charge state of 2–5 and an abundance threshold of more than 90 counts (cps). Former target ions were excluded for 15 s. IDA rolling collision energy (CE) parameters script was used for automatically controlling the CE.

#### 4.5.3. Data Analysis and Quantification

The mass spectrometry data obtained were processed using PeakView^®^ 2.2 Software (SCIEX Foster City, CA, USA) and exported as mgf files. Proteomic data analyses were performed by using 4 search engines (Mascot Server v.2.6.1, OMSSA, X!Tandem and Myrimatch) and a target/decoy database built from sequences in the *Homo sapiens* proteome at Uniprot Knowledgebase. All search engines were configured to match potential peptide candidates to recalibrated spectra with mass error tolerance of 10 ppm and fragment ion tolerance of 0.02 Da, allowing for up to two missed tryptic cleavage sites and a maximum isotope error (13C) of 1, considering fixed MMTS modification of cysteine and variable oxidation of methionine, pyroglutamic acid from glutamine or glutamic acid at the peptide N-terminus. Score distribution models were used to compute peptide-spectrum match *p*-values [114], and spectra recovered by a false discovery rate (FDR) ≤ 0.01 (peptide-level) filter were selected for quantitative analysis. Differential regulation was measured using linear models [115], and statistical significance was measured using q-values (FDR). All analyses were conducted using software from Proteobotics S.L. (Madrid, Spain). Functional analyses were performed with Ingenuity Pathway Analysis, IPA (Qiagen, Hilden, Germany). The mass spectrometry proteomics data have been deposited to the ProteomeXchange Consortium (http://proteomecentral.proteomexchange.org) via the PRIDE partner repository [116] with the ID PXD019924. 

### 4.6. Data Analysis and Machine Learning

#### 4.6.1. Descriptive and Inferential Statistics

Most of the clinical and analytical data were not normally distributed, and even when several transformation techniques were applied the homogeneity of variance requirement was rarely met. On the other hand, non-parametric statistics were also not applicable, as the groups rarely followed the same distribution and it often was very complex (multimodal). For that reason, *p*-values were calculated using permutation techniques [117,118]. Permutation techniques, as classical statistical tests, assume that the null hypothesis (H_0_) is true, in other words, there are no differences between groups and thereby the labels (individual conditions: Control, CCA or PDAC) are exchangeable. The algorithm makes all possible rearrangements of labels on the data and then computes how many times the differences between the groups are equal or more extreme than the observed ones, that translated into probability, is the definition of *p*-value. This technique avoids also the unbalanced design of our experiment. Data are expressed as means ± SD.

#### 4.6.2. Machine-Learning Pipeline

##### Multivariate Analysis

Multivariate analyses, including principal component analysis (PCA) [119] and discriminant analysis of principal components (DAPC) of metabolomic and proteomic data were performed as previously described [59,119].

##### Data Imputation

Data derived from metabolomic and proteomic studies were used to carry out artificial intelligence to uncover possible patterns that may help in the diagnosis of these pathologies. To this end, first, missing data must be deleted or imputed. The sample size was not large enough to delete the missing data, so data was imputed using R software Version 3.6.2 [120] package VIM Version 5.1.1 [121], as previously described in similar studies [122].

##### Synthetic Data Generation

Once the analytical data were generated, using the mean**,** standard deviation and correlation information, the synthetic data was generated with MASS package v7.3-51.4 [62]. At this point no distribution-based methods were used regarding artificial intelligence methods, for that reason, the modification of the media or the shape of distribution does not affect the outcome. Integer data was generated for proteomic analysis, whereas decimal data was generated for metabolomic data. Scripts for synthetic data generation can be accessed at: https://github.com/HepatologiaCIMA/Urman_and_Herranz_etal_2020.

##### Feature Selection

Three methodologies were used for feature selection, AUC, Random Forest (RF) and DAPC. In the case of AUC, AUC was computed for every variable using CARET package Version 6.0-86 [123] for the synthetic data. The CARET package was also used for RF analyses. AUC, RF and DAPC methodologies were independently used to select the minimum number of features (within a range of 3 to 10 variables) that best explained the separation between groups.

##### Artificial Intelligence Analysis

The sets of features (variables) were imputed into four algorithms from the CARET package (v6.0-86), neural networks (NN) [124,125,126], Bayesian general linear model [127], C5.0 and RF [128]. In the feature selection step, RF is used to select features, whereas in the training step it is used as a classification algorithm. We have included RF as a typical algorithm used when the dimensionality of the data is extremely large compared to the measures. The algorithm with highest AUC was then statistically tested. Five types of custom tests to evaluate the prediction capacity of our model were applied. Test 1, aimed at calculating the probability of randomly obtaining the same result, consists of reordering the labels (identity of the samples) of the real data to obtain the probability of getting the same result by chance. It can be interpreted as the chance of randomly predicting the data as good as the model does. In our analyses it revealed that this probability was negligible, even for proteomic data with a low number of samples, and always showed a value of *p* < 0.001. Test 2, aimed at computing the importance of each variable for that specific model, randomly reorders thousands of times its values across the whole cohort of patients and then applies the model. The probability of getting a result as good or better than the original one is computed if that variable is random. It can be interpreted as how an error in the analytical measurement of a variable can affect the prediction. The application of this test indicated that more features needed to be selected for the model to perform robustly in the lipidomic analysis than in the proteomic analysis. Test 3, aimed at computing the fitness of the model, randomly reorders all the variables to count how many times the model can achieve a result as good or better than original one with unstructured data. It can be interpreted as noise prediction or background prediction. This test demonstrated with a *p* < 0.001 that the data was structured in both the lipidomic and proteomic sets. For Test 4, some of the variables can be very predictive, so reordering only one of them may be compensated by the others. The aim of this test is to compute, in the selected model, the importance of a single variable in the prediction of the outcome, reordering all the other variables randomly. It can be interpreted as the capacity of the variable to predict the outcome in the presence of noise. We found that none of the selected features alone were able to accurately classify the samples. In Test 5, the synthetic data generated is more abundant than the validation set and considering that we used sample measures to simulate population data, one may think that we are overfitting the model for a given sample and that the prediction will have nothing to do with the reality of the data [129]. This test randomly shuffles the labels and then it computes synthetic data and subsequently tries to elaborate a predictive model for shuffled data. Then, using permutations test, we evaluate the differences in shuffled vs. real data AUCs. Through this approach we can assess the tendency of the synthetic data to overfit the model. The graphic representation of our NN analyses was made using the NeuralNetTools package as previously described [126]. Scripts for the built NN models (for the selected features) and the trained NN models described in this study are available in this link: https://github.com/HepatologiaCIMA/Urman_and_Herranz_etal_2020.

## 5. Conclusions

The etiological diagnosis of biliary strictures is still a clinical challenge. Bile, collected during the little invasive ERCP procedure, may be a good source of biomarkers to identify the presence of neoplastic disease. Over the past fifteen years several studies have performed high-throughput metabolomic and proteomic studies of bile obtained from patients with biliary obstruction and different cholangiopathies. Although some potential biomarkers, i.e., lipid species and proteins, have been identified, the high variability among samples, together with the high cost of performing “omic” analyses in large cohorts of patients, have hindered the identification of robust biomarkers. In this work, we have revisited the metabolome and proteome of human bile from patients with benign cholangiopathies and malignant biliary strictures. We are aware of some limitations affecting this study, including its preliminary nature, its case-control and single-center design, and the lack of an independent validation cohort for our features and algorithm combinations. Furthermore, we did not include in our study bile samples from patients with primary sclerosing cholangitis, a predisposing condition for CCA development. Given the heterogeneity of both benign and malignant biliopancreatic conditions, future “omic” studies should focus on more homogeneous groups of patients. For instance, a recent quantitative proteomic analysis of bile included only patients with extrahepatic CCA and controls without biliary disease [66]. Targeted analyses of the lipids and proteins selected in this study, rather than shotgun lipidomics and proteomics, may also provide additional robustness to our model. Despite these considerations, here we have performed what we believe is the most comprehensive characterization of the human bile lipidome reported so far. The analyses that have been carried out, together with our complementary ^1^H-NMR study, identified alterations in metabolites that may be linked to the biliary and pancreatic malignant processes. Similarly, the proteomic profile used here also identified changes in protein levels that may capture molecular alterations evolving in tumor cells. Nevertheless, looking at the complexity of the complement of metabolites and proteins present in bile, and their interindividual variability, we understood that more complex analytical tools would be needed to expose useful biomarkers. Thus, we decided to implement alternative methods, including machine-learning approaches for the generation of synthetic data to enlarge our experimental data set, tested different alternative methods for biomarker selection (DAPC, AUC and RF analyses), and assayed different algorithms to unravel the complex patterns and interrelations existing among metabolites or proteins that may be the key for sample discrimination. We came up with a combination of lipids and proteins (features) that when analyzed with NN provided a predictive model for the eventual classification of patients with biliary strictures. Our present findings lend further support to the potential of machine intelligence for the development of predictive models in the analysis of complex biological samples such as human bile. Nevertheless, the accuracy of the specific biomarkers identified here using artificial intelligence tools will need to be validated with real data from independent cohorts of patients. Finally, in future studies it would also be interesting to test the combined performance of bile proteomic and metabolomic biomarkers for patient classification in the context of biliopancreatic diseases.

## Figures and Tables

**Table 1:** Demographic and clinical characteristics of the study cohort.

Variables	Benign Biliary Conditions (*n* = 36)	CCA (*n* = 36)	PDAC (*n* = 57)	*p* Value *
Age, median (years) ± SD	66 ± 19	74 ± 12	71 ± 12	^a^*p =* 0.05, ^b^ *p =* 0.09
Gender (Male/Female)	19/17	17/19	25/32	*p* = 0.718
Location of biliary stenosis (Distal/Hilar/Intrahepatic)	10/0/1	18/15/3	57/0/0	
Operated stenosis **	1 (9.1%)	14 (38.9%)	16 (28%)	
Stage IV (AJCC Pronostic Group ***)	NA	8 (22.2%)	15 (26.3%)	
Body Mass Index (kg/m^2^)	27.28 ± 4.56	25.26 ± 4.65	25.86 ± 4.96	^a^*p* = 0.067, ^b^ *p =* 0.169
Bilirrubin (mg/dL)	3.18 ± 3.10	9.05 ± 7.78	10.79 ± 7.11	^a^*p* = 0.00019, ^b^ *p =* 0.00000037
Albumin (g/dL)	3.69 ± 0.47	3.29 ± 0.57	3.46 ± 0.47	^a^*p* = 0.0029, ^b^ *p =* 0.029
GGT (U/L)	609 ± 517	1013 ± 678	1116 ± 724	^a^*p* = 0.0078, ^b^ *p =* 0.00083
INR	1.13 ± 0.17	1.14 ± 0.22	1.13 ± 0.15	^a^*p* = 0.8, ^b^ *p =* 0.98
Total cholesterol (mg/dL)	171 ± 48	225 ± 82	233 ± 107	^a^*p* = 0.0018, ^b^ *p =* 0.0026
Triglycerides (mg/dL)	138 ± 81	169 ± 105	178 ± 81	^a^*p* = 0.187, ^b^ *p =* 0.031
PNI ****	44.80 ± 6.74	41.41 ± 6.81	41.82 ± 5.95	^a^*p* = 0.042, ^b^ *p =* 0.033
High CA19-9 (>37 U/L) *****	10 (27.8%)	24 (66.7%)	46 (80.7%)	^a^*p* = 0.578, ^b^ *p =* 0.065

* a = CCA vs. Benign biliary conditions, b = PDAC vs. Benign biliary conditions. ** 31 (29.8%) patients with biliary stenosis underwent surgery. *** AJCC: American Joint Committee on Cancer staging system; NA: Not applicable; **** PNI: Prognostic Nutritional Index. ***** Serum CA19-9 was measured in 110 (85.3%) patients.

## Supplementary Materials

The following are available online at https://www.mdpi.com/2072-6694/12/6/1644/s1, Figure S1: Correlation between serum levels of bilirubin or GGT and BAs levels in bile in control, CCA and PDAC patients, Figure S2: Graphical representation of neural network analysis of selected lipidomic biomarkers, Figure S3: PCA and DAPC analyses of bile proteomics data, Figure S4: Graphical representation of neural network analysis of selected proteomic biomarkers, Table S1: Heatmap of the lipidomic analysis of bile, Table S2: AUC, sensibility and specificity of metabolites selected by DAPC analysis of lipidomic data, Table S3: List of differentially represented proteins in bile from control and CCA patients, Table S4: List of differentially represented proteins in bile from control and PDAC patients.
